# Supplementary material for: Comparison of storage and lignin accumulation characteristics between two types of snow pea
Source: PLoS One. 2022 Jul 1;17(7):e0268776. doi: 10.1371/journal.pone.0268776 (PMC9249198; doi:10.1371/journal.pone.0268776)
Supplement: Attachment — Submitted filename: PONE-D-21-40678_reviewer.pdf [file pone.0268776.s003.pdf]

PLOS ONE

Senescence and lignin accumulation lead to hardening of snow pea during postharvest storage

--Manuscript Draft--

**Manuscript Number:**	PONE-D-21-40678
**Article Type:**	Research Article
**Full Title:**	Senescence and lignin accumulation lead to hardening of snow pea during postharvest storage
**Short Title:**	Senescence and lignin accumulation lead to hardening of snow pea
**Corresponding Author:**	Xuerui Li<br>Yunnan Academy of Agricultural Sciences<br>Kunming, CHINA
**Keywords:**	Snow pea, metabolic rate, nutrient consumption, lignification, texture properties
**Abstract:**	Snow pea is a very important vegetable, and its postharvest storage characteristics vary with species. Few studies on the differences in its storage characteristics are available. In this study, postharvest changes in metabolic rate (respiration rate and water loss rate), membrane permeability (relative conductivity), nutrient contents (total sugar, amino acids, starch), lignin, cellulose, texture properties , and their relationship were analyzed in large sweet broad peas and small snow peas . On the 8 th day of storage, we found that the respiration rate and water loss rate were increased, total sugars and total amino acids were decreased significantly in these two legume vegetables, and that metabolic rate was slower with less nutrients consumed in large sweet broad peas than in small snow peas. Througout the 8-day whole storage, the lignin and cellulose contents were always lower in large sweet broad peas than in small snow peas. With the increasing storage time, small snow peas were more susceptible to lignification and fibrosis, which was observed in their texture properties. At the late stage of storage, the taste of large sweet broad peas was better than that of small snow peas. In conclusion, the storage period of large sweet broad peas was longer than that of the small snow peas, and its lignification degree was lower than that of the small snow peas. Meanwhile, senescence and lignin accumulation led to hardening of snow pea during postharvest storage. Our findings provide a theoretical reference for improving the postharvest storage quality of snow pea and extending the shelf life.
**Order of Authors:**	Xuerui Li
	Jin Xiang Wang
	Yun Hui Qu
	Yi ping Li
	Humaira Yasmin
	Muhammad Abdul Rehman Rashid
	Hongmei Pu
	Li Juan Yu
	Hong Li
**Additional Information:**	
**Question**	**Response**
**Financial Disclosure**<br><br>Enter a financial disclosure statement that describes the sources of funding for the work included in this submission. Review	The present research got supportion from the Yunnan Li Puwang Expert Workstation (202005AF50007). Yu Lijuan and Li Xuerui are the main accomplisher of this project,they design the study, collect and analyse dates,prepare the manuscripts; it also got supportion from Cultivation of Agricultural Product Processing Team (202002AE320007-03).Li Hong is the project director, he design this study.

Powered by Editorial Manager® and ProduXion Manager® from Aries Systems Corporation

the submission guidelines for detailed requirements. View published research articles from *PLOS ONE* for specific examples.<br><br>This statement is required for submission and **will appear in the published article** if the submission is accepted. Please make sure it is accurate.<br><br>**Unfunded studies**<br>Enter: *The author(s) received no specific funding for this work.*<br><br>**Funded studies**<br>Enter a statement with the following details:<br>• Initials of the authors who received each award<br>• Grant numbers awarded to each author<br>• The full name of each funder<br>• URL of each funder website<br>• Did the sponsors or funders play any role in the study design, data collection and analysis, decision to publish, or preparation of the manuscript?<br>• **NO** - Include this sentence at the end of your statement: *The funders had no role in study design, data collection and analysis, decision to publish, or preparation of the manuscript.*<br>• **YES** - Specify the role(s) played.<br><br>* typeset	
**Competing Interests**<br><br>Use the instructions below to enter a competing interest statement for this submission. On behalf of all authors, disclose any competing interests that could be perceived to bias this work—acknowledging all financial support and any other relevant financial or non-financial competing interests.<br><br>This statement is required for submission and **will appear in the published article** if the submission is accepted. Please make sure it is accurate and that any funding sources listed in your Funding Information later in the submission form are also	None of our co-authors has conflict of interest to declare.

Powered by Editorial Manager® and ProduXion Manager® from Aries Systems Corporation

declared in your Financial Disclosure statement.<br><br>View published research articles from *PLOS ONE* for specific examples.<br><br>**NO authors have competing interests**<br><br>Enter: *The authors have declared that no competing interests exist.*<br><br>**Authors with competing interests**<br><br>Enter competing interest details beginning with this statement:<br><br>*I have read the journal's policy and the authors of this manuscript have the following competing interests: [insert competing interests here]*<br><br>* typeset	
**Ethics Statement**<br><br>Enter an ethics statement for this submission. This statement is required if the study involved:<br><br>• Human participants<br>• Human specimens or tissue<br>• Vertebrate animals or cephalopods<br>• Vertebrate embryos or tissues<br>• Field research<br><br>Write "N/A" if the submission does not require an ethics statement.<br><br>General guidance is provided below. Consult the submission guidelines for detailed instructions. **Make sure that all information entered here is included in the Methods section of the manuscript.**	no

**Format for specific study types**<br><br>**Human Subject Research (involving human participants and/or tissue)**<br>• Give the name of the institutional review board or ethics committee that approved the study<br>• Include the approval number and/or a statement indicating approval of this research<br>• Indicate the form of consent obtained (written/oral) or the reason that consent was not obtained (e.g. the data were analyzed anonymously)<br><br>**Animal Research (involving vertebrate animals, embryos or tissues)**<br>• Provide the name of the Institutional Animal Care and Use Committee (IACUC) or other relevant ethics board that reviewed the study protocol, and indicate whether they approved this research or granted a formal waiver of ethical approval<br>• Include an approval number if one was obtained<br>• If the study involved *non-human primates*, add *additional details* about animal welfare and steps taken to ameliorate suffering<br>• If anesthesia, euthanasia, or any kind of animal sacrifice is part of the study, include briefly which substances and/or methods were applied<br><br>**Field Research**<br><br>Include the following details if this study involves the collection of plant, animal, or other materials from a natural setting:<br>• Field permit number<br>• Name of the institution or relevant body that granted permission	
**Data Availability**<br><br>Authors are required to make all data underlying the findings described fully available, without restriction, and from the time of publication. PLOS allows rare exceptions to address legal and ethical concerns. See the PLOS Data Policy and FAQ for detailed information.	Yes - all data are fully available without restriction

A Data Availability Statement describing where the data can be found is required at submission. Your answers to this question constitute the Data Availability Statement and **will be published in the article**, if accepted.<br><br>**Important:** Stating 'data available on request from the author' is not sufficient. If your data are only available upon request, select 'No' for the first question and explain your exceptional situation in the text box.<br><br>Do the authors confirm that all data underlying the findings described in their manuscript are fully available without restriction?	
**Describe where the data may be found in full sentences. If you are copying our sample text, replace any instances of XXX with the appropriate details.**<br><br>• If the data are **held or will be held in a public repository**, include URLs, accession numbers or DOIs. If this information will only be available after acceptance, indicate this by ticking the box below. For example: *All XXX files are available from the XXX database (accession number(s) XXX, XXX.).*<br>• If the data are all contained **within the manuscript and/or Supporting Information files**, enter the following: *All relevant data are within the manuscript and its Supporting Information files.*<br>• If neither of these applies but you are able to provide **details of access elsewhere**, with or without limitations, please do so. For example:<br><br>*Data cannot be shared publicly because of [XXX]. Data are available from the XXX Institutional Data Access / Ethics Committee (contact via XXX) for researchers who meet the criteria for access to confidential data.*<br><br>*The data underlying the results presented in the study are available from (include the name of the third party*	All relevant data are within the manuscript and its Supporting Information files.

and contact information or URL).<br>• This text is appropriate if the data are owned by a third party and authors do not have permission to share the data.<br><br>* typeset	
Additional data availability information:	

Powered by Editorial Manager® and ProduXion Manager® from Aries Systems Corporation

Manuscript
Click here to access/download;Manuscript;001 Lixuerui20211225.docx

Senescence and lignin accumulation lead to hardening of snow pea during postharvest storage

[1]Li, X.R., [2]Wang, J.X., [1]Qu, Y.H., [1]Li, Y.P., [3]Humaira,Y., [3]Muhammad, S., [1]Pu, H.M., [1]*Yu, L.J., [1]*Li, H.

[1]Agro-products Processing Research Institute, Yunnan Academy of Agricultural Sciences, Kunming, Yunnan, China, 650205; [2]School of Life Sciences, Datong University, Datong Shanxi 037009, China. [3]Department of Biosciences, COMSATS University Islamabad (CUI), Park Road, Islamabad 45550, Pakistan

Corresponding author: Yu Lijuan, Agro-products Processing Research Institute, Yunnan Academy of Agriculutral Sciences, 650205 Kunming, P.R. China. Email: ylj@yaas.org.cn;Li Hong, Agro-products Processing Research Institute, Yunnan Academy of Agriculutral Sciences, 650205 Kunming, P.R. China. Email: ynveg@163.com;

Abstract: Snow pea is a very important vegetable, and its postharvest storage characteristics vary with species. Few studies on the differences in its storage characteristics are available. In this study, postharvest changes in metabolic rate (respiration rate and water loss rate), membrane permeability (relative conductivity), nutrient contents (total sugar, amino acids, starch), lignin, cellulose, texture properties, and their relationship were analyzed in large sweet broad peas and small snow peas. On the 8th day of storage, we found that the respiration rate and water loss rate were increased, total sugars and total amino acids were decreased significantly in these two legume vegetables, and that metabolic rate was slower with less nutrients consumed in large sweet broad peas than in small snow peas. Througout the 8-day whole storage, the lignin and cellulose contents were always lower in large sweet broad peas than in small

snow peas. With the increasing storage time, small snow peas were more susceptible to lignification and fibrosis, which was observed in their texture properties. At the late stage of storage, the taste of large sweet broad peas was better than that of small snow peas. In conclusion, the storage period of large sweet broad peas was longer than that of the small snow peas, and its lignification degree was lower than that of the small snow peas. Meanwhile, senescence and lignin accumulation led to hardening of snow pea during postharvest storage. Our findings provide a theoretical reference for improving the postharvest storage quality of snow pea and extending the shelf life.

Key words: Snow pea, metabolic rate, nutrient consumption, lignification, texture properties

1 Introduction

Snow pea (*Pisum sativum* L. var. macrocarpon Ser.) is grown worldwide, extensively in northern USA, Europe, Canada, Russia, and China (Griga *et al.*, 1990; Guindon *et al.*, 2021). Due to the delicious flavor and abundant nutrients, its tender shoot, seed, and pod are consumed as vegetables, especially the tender pod (Shi *et al.*, 2014). Its pod is one of the most important and popular leguminous vegetable because of high phytonutrients including amino acids, insoluble fibers, and vitamins (Pariasca *et al.*, 2001). However, its pod is is highly perishable and ripened within a few days with a relatively short shelf-life, thus restricting its market potential.

Due to the high water content, strong metabolism, and microorganism reproduction, it is easy for pods to lose water, wrinkle, and then resulting in nutrient loss even deterioration during storage (Kader, 1992; Bijay *et al.*, 2020). Kader *et al.* (1992) have found that precooling and storing at near 0°C could extend the shelf life of pea pod. Kumar *et al.* (2016) have reported that cumin essential oil is preservative to extend shelf life of *Pisum sativum*. Nasef *et al.* (2018) have revealed that microperforated polypropylene bags with 12 microholes are suitable to preserve pea pod, thus ~~extend it~~ retail period. However, available information has been reported on the optimization of pea storage conditions and the information on the deterioration

mechanism in pea is relatively scarce. El-hamahmy *et al*. (2017) have suggested that decay, shriveling, and reduction in vitamin C, and sugar contents are the primary factors limiting storage quality of snow pea pods.

Lignification is common in plants, and lignin can guarantee the integrity of cell wall, enhance the hardness and toughness of stem, provide plants with the internal mechanical support so that plants can resist abiotic and biotic stress (Peter *et al*., 2004; Zhu *et al*., 2021). The lignification not only affects the taste and quality, but also limits the storage and transport of postharvest horticultural products. For example, lignin content inmaize exhibits an extremely significant negative correlation with edible quality (Melida *et al*., 2015). The pea pods become hard and coarse with more fiber, which affects the taste of the legumes.

In the present study, the changes in metabolic rate and nutrient contents were investigated to reveal deterioration mechanism in super snap peas after harvest. The metabolic rate indexes were significantly increased including the respiration rate, water loss rate, electrical conductivity. The nutrient contents indexes were rapidly declined including total sugar, total amino acids, and starch. Meanwhile, lignin and cellulose contents were ascended. Further, the texture properties such as hardness and chewiness changed. These results imply that lignification in snow pea may cause the change in texture properties, thus affecting its taste and quality. The aim of this study was to evaluate the storage characteristics of legume vegetables and lay the foundation for further research on related lignification and preservation technologies.

2 Materials and methods

2.1 Materials

Two varieties of snow pea including large sweet broad pea and small snow pea were purchased from the vegetable supermarket on the agricultural product display platform of Agro-product Processing Research Institute, Yunnan Academy of Agricultural Sciences. Freshly picked pods were selected, and they were intact, free from pests and mechanical damage with similar size, maturity degree, and plumpness.

2.2 Assessments

To investigate the physiological and biochemical changes during storage, pods stored after 2, 4, 6, and 8 days were used as test samples. To avoid the possible error, the head and tail of selected pods were removed, and the middle part of pods was used for index measurement.

Respiration rate. Respiration rate was measured with SY-1022 Fruit and Vegetable Respiro meter. First, samples were respectively put into the respiratory chamber, after standing for 0.5 h, the respiratory intensity of the sample was caculated according to the change of CO_2 concentration in the respiratory chamber before and after sample placement.

Water loss rate. The water loss rate was determined according to the change in the sample weight after harvest and during storage (Alvarez *et al*., 2002). The initial weight of the sample was recorded as M0, the weight of each sample at sampling time points was recorded as M. The water loss rate was computed according to the following formula:

$$Water\ loss\ rate\ (\%)\ = (M0 - M)\ /M0.$$

Relative conductivity. The samples were cut into small-sized pieces, and soaked in 20 mL of distilled water for 1 h. Afterwards, the initial electronic conductivity (C0) was recorded, and then the sampls were boiled for 30 minutes and cooled to record the electronic conductivity value (C1). Finally, the electronic conductivity was computed as follows:

$$Relative\ conductivity\ (\%)\ = (C0/C1) * 100\%$$

Total sugar content. First, the total sugar was extracted, A total of 2 g sample was added into 20 mL of distilled water, and then was boiled for 30min. The standard curve of glucose was constructed, y=0.0037x+0.0928. Then it was determined according to the modified anthrone colorimetric method (Nasef *et al*., 2018). The absorbance value at 630 nm was measured. The total sugar content in the sample was calculated by using the following formula:

$$Total\ sugar\ content\ (\%)\ = \ (C * V1 * D)/\ (W * V2 * 106)\ * 100\%$$

Whereas, C=sugar concentration (μg); V_1=total volume of sugar extract (mL); D=dilution factor; W=total weight of sample (g); V_2=volume of sugar used for the

determination (mL).

Total Amino acid content. The amino acid content was determined according to the improved ninhydrin colorimetric method (Song *et al*., 2010). A total of 5 g sample was added into 50 mL of distilled water and 5g activated carbon, and then was boiled, and filtered. Afterwards, 2 mL filtrate was added into distilled water (2 mL), 2% ninhydrin solution (1 mL), and phosphate buffer (1 mL), and boiled for 15 min. After cooling down, absorbance at 570 nm was measured.

Starch content. The starch content was determined with the plant starch content kit (Suzhou Keming Biotechnology Co., Ltd.). After measurement of the absorbance at 620 nm, starch content was calculated according to the following formula:

$$Starch\ content\ (mg/g\ fresh\ weight)\ = [(A + 0.0295)\ * V1]/2.936/(W * V1/V2)$$

Where A is the absorbance value; V_1 is the volume of the sample added to the reaction system; V_2 is the volume of extract added; W is the sample weight.

Lignin content. The sulfuric acid method (Maekawa *et al*., 1989) was used to determine the lignin content. The initial weight of the sample was recorded as M0. The initial sample was added into 4 mL sulfuric acid (72%) and 200 mL distilled water, refluxed at ~~100□~~ for 1 h, and filtered. ~~Andthen,~~ the filter residue was washed with hot distilled water, and dried completely to a constant weight (M). The lignin content was calculated as follows:

$$Lignin\ content\ (\%)\ =\ (M/M0)\ *\ 100.$$

Cellulose content. Cellulose content was determined by the method of acidity washing. The samples were added into 20 mL of cetyl trimethyl ammonium bromide. The remaining procedures were simialr to those for the determination of lignin.

Texture properties. The hardness, cohesiveness, springiness, gumminess, and chewiness were measured by the TMS-TOUCH texture analyzer equipped with a cylindrical detection probe (35 mm diameter). The test parameters were set as follows: the load unit of 200 N (Newton), the target deformation of 50%, the test speed of 60 mm/s, and the rising height (from the sample) of 10 mm, the initial force of 0.1 N, and the residence ~~timeof~~ 0 s between two extrusion cycles. The measurement was repeated

10 times.

2.3 Statistical analysis

All data were presented as the mean ± standard error (SE). The statistical difference between groups was analyzed through one-way analysis of variance (ANOVA) in SPSS software. $P<0.05$ was considered as statistically significant.

3. Results and Discussion

3.1 Respiration rate and water loss

Water is essential to ensure the freshness of fruits and vegetables. When water loss exceeds 5%, fruits and vegetables usually begin to wilt and lose the freshness (Jin *et al.*, 2007). However, due to the strong respiration and transpiration, legumes are prone to lose water and wilt, and deteriorate. Hence, the respiration rate and water loss rate were examined in the present study (Fig 1). As shown in Fig 1A, the respiration rate was dramatically increased by 55.38% (from 66.54 to 103.39 mg • $kg^{-1} \cdot h^{-1}$) for large sweet broad pea and by 139.75% (from 30.77 to 73.77 mg·$kg^{-}1 \cdot h^{-1}$) for small snow peas after 8-day storage, respectively. This indicated that the respiration rate of the small snow peas was stronger. In addition, it was observed that the water loss rate of large sweet broad peas increased from 13.79% to 32.56%, and that of small snow peas increased from 15.82% to 36.03% (Fig1B). The water loss rate of both types of peas increased during storage, while compared to large sweet broad peas, small snow peas is more likely to lose water. Above results indicated that respiration intensity of legumes increased with the extension of storage, which might closely related with water loss.

3.2 Relative conductivity

Relative conductivity is an important indicator for estimating the degree of destruction of cell membrane structure in fruits and vegetables in the course of storage (Liu *et al.*, 2016). Therefore, the current study analyzed the changes in relative conductivity of two snow pea during storage. As shown in Fig 2, the relative conductivity was increased by 30.68% (from initial 37.68% to 49.24%) for large sweet broad peas and by 47.33% (from 34.73% to 51.17%) for small snow peas after storing

for 8 days. This indicated that the cell membrane structure ~~in postharvest~~ small snow peas is more drastically destructed than large sweet broad peas during storage.

3.3 Total sugar, total amino acid, and total starch content

Sugar, amino acids, and starch are the main nutrients in legume vegetables, ~~and they directly determine~~ nutritional and edible values of legume vegetables. However, these nutrients were usually declined with the extension of storage (Ishaq *et al*., 2009). Hence, in the current study, total sugar, total amino acids, and starch were investigated (Fig 3). As shown from Fig 3A and 3B, both the total sugar and total amino acid decreased with the extension of storage. The total sugar content declined relatively smooth from 4.42% to 3.35% in large sweet broad peas, whereas that in small snow pea declined sharply from 3.96% to 1.10% (Fig 3A). Thus, after 8-day storage, total sugar content of small snow pea was significantly lower than that of large sweet broad peas.

As shown in Fig 3B, the change in amino acid content was consistent with that in total sugar content, which decreased respectively by 7.5% in large sweet broad peas and 14.1% in small snow peas. Although, there was a rise from 19.72 mg·g^{-1} FW at the day 0 of storage to 29.14 mg·g^{-1} FW at day 2 for large sweet broad pea, which might be caused by the ~~after-ripening~~. The total amino acid content ~~fallen~~ from 29.14 mg·g^{-1} FW at the 2th day to 18.24 mg·g^{-1} FW at the ~~final~~ of storage in large sweet broad peas. Comparatively, the the total amino acid content declined smoothly in small snow peas, which decreased from 28.78 mg·g^{-1} FW at the intinal to 24.72 mg·g^{-1} FW at the ~~final.~~

Also, the change in starch content was analyzed (Fig 3C). The starch content of large sweet broad peas increased from initial 14.19 mg·g^{-1} FW to 17.45 mg·g^{-1} FW on day 8, which might be attributed by after-ripening. While, the starch content of small snow peas decreased by 7.06 mg·g^{-1} FW from 18.47 mg·g^{-1} FW after harvest to 11.41mg·g^{-1} FW on the 8th day of storage. According to above results, nourishments including the total sugar and the total amino acid was delpeted in both two varities after harvest. And it was indicated that the nutrients of small snow peas were consumed more quickly than large sweet broad peas.

3.4 Lignin and cellulose content

Lignin and cellulose, as important components of plant secondary cell wall, participate in various physiological processes, excepically in the morphological development of plants (Terrett *et al.*, 2019). Lignification can ensure the integrity of cell wall, enhance the hardness and toughness of stem, and provide plants with the internal mechanical support so that plants can resist abiotic and biotic stresses (Peter *et al.*, 2004). However, the accumulation of lignin and cellulose not only affects the taste and quality of fruits and vegetables, but also limits their postharvest storage and transportation. It has been reported that fruits and vegetables become hardened due to the increased lignin and cellulose during storage or under stress conditions (Kano *et al.*, 1994).

In the present study, lignin content in both types of peas significantly increased with the extension of the storage (Fig 4A). Although, the lignin content in large sweet broad peas raised from 10.60 mg·~~g-1~~ to 33.57 mg·~~g-1~~ after 8-day storage, but the lignin content in large sweet broad peas was always lower than that in small snow peas. This indicated that the small snow ~~peaswere~~ more easily lignified with the increasing storage ~~time~~. The cellulose ~~compare~~ between two types of peas exhibited the similar regularity as lignin content (Fig 4B). In large sweet broad peas, the cellulose content increased from 35.65 mg·g^{-1} to 37.48 mg·g^{-1} in the first 4 days of storage, and then ~~was~~ decreased to 31.01 mg·g^{-1} in the last 4 days of storage, which might be due to the after-ripening post harvest. However, the cellulose content increased from 39.67 mg·g^{-1} to 46.06 mg·g^{-1} after storage in the small snow peas during 8-day storage. In the processing of storage, the cellulose content in large sweet broad peas was also always lower than that in small snow peas.

3.5 Texture characteristics

With the increase in lignin and cellulose contents during the storage, the textural properties of fruits and vegetables ~~change~~ greatly. The textural properties of fruits and vegetables are also important for evaluating their quality and economic values (Rissolo

et al., 2014). Usually, the texture profile analysis (TPA) method was used to simulate the chewing motion of human teeth to analyse the textural properties, in which the sample was compressed twice to test ~~itscohesiveness~~, springiness, gumminess, and chewiness. This texture evaluation method reduced the subjective evaluation error to a certain extent (Broolfield *et al.*, 2011).

In this study, the TPA method was used to analyze ~~the~~ changes in ~~texture~~ textural properties of the two types of peas during storage. The results indicated that the cohesiveness of two types of peaswas increased by 21.83% from 0.20 to 0.24 for large sweet broad peas and increased by 121.63% for small snow peas from 0.17 to 0.39. This corresponded exactly to the water loss rate, and a large amount of water loss caused the internal cohesiveness to increase.

The springiness of the two types of peas showed an upward trend until the 6th day of storage, increasing from 3.023 to 4.06 mm for large sweet broad peas, and from 2.605 to 3.7 mm for small snow peas, and then springiness declined relatively smoothly in both types of peas. Overall, in 8-day storage, springiness increased by 8.9% for large sweet broad peas and by 33.05% for small snow peas.

The gumminess and chewiness of the two types of peas showed a rapid upward trend during 8-day storage. In large sweet broad peas, the gumminess increased by 57.53% from 1.00 to 2.73 N, and the chewiness increased by 209.13% from 3.021 to 9.30 N. On the 8th day, gumminess and chewiness values of large sweet broad peas were slightly decreased, which was smilar to springiness in large sweet broad peas, and this decrease might be attributed to the consumption of nutrients in the late storage stage. In small snow peas, the gumminess increased by 245.90% from 1.07 to 3.70 N, and the chewiness increased by 375.53% from 2.73 to 12.97 N. ~~Compare~~ to the large sweet broad peas, the rising trend of chewiness in small snow peas was more obvious.

4 Conclusion

The study revealed that small snow peas had a fast metabolic rate and consumed more nutrients than large sweet broad peas after harvest, resulting in the increased

membrane permeability and greater perishability. At the same time, the lignification degree of small snow peas was higher than that of large sweet broad peas after harvest, which affected their taste and shelf life. These results were consistent with the change in their texture characteristics. The water loss rate of small snow peas during storage was more serious than that of large sweet broad peas, which caused small snow peas to lose their crispness. In addition, the consumption of nutrients and the lignification might be responsible for the increasing springiness and chewiness in snow pea. Overall, large sweet broad peas had a longer shelf life and a lower lignification degree than small snow peas. Future research is suggested to focus on the lignification of snow pea during storage and the exploration of preservation methods so as to alleviate the lignification of snow pea. This study provides theoretical basis for the preservation of legume vegetables.

Acknowledgments

The present research got supportion from the Yunnan Li Puwang Expert Workstation (202005AF50007), Cultivation of Agricultural Product Processing Team (202002AE320007-03). Great gratitude goes to linguistics professor Ping Liu (Huazhong Agricultural University, Wuhan, China) for her work at English editing and language polishing. None of our co-authors has conflict of interest to declare.

References

Alvarez, M., Canet, W., Lopez, M. 2002. Influence of defomation rate and degree of compression on textural parameters of potato and apple tissues in texture profile analysis~~[J]~~. European Food Research and Technology 215: 13-20.

Bijay, K., Achintya, M., Rakesh, P. and Priyanjani, S.A. 2020 . Review of physiological and biochemical changes related to ripening along with post harvest handling and treatments of Sapota. Journal of Pharmacognosy and Phytochemistry, 9(4): 2030-2035.

Broolfield, P.L., Nicoll, S., Gunson, F.A., Harker, F.R., Wohlers, M. 2011. Sensory evaluation by small postharvest teams and the relationship with instrumental measurements of apple texture~~[J]~~. Postharvest Biology & Technology 59(2): 179-186.

El-hamahmy, M.A.M., ElSayed, A.I., Odero, D.C. 2017. Physiological effects of hot water dipping, chitosan coating and gibberellic acid on shelf-life and quality assurance of sugar snap peas (*Pisum sativum L. var. macrocarpon*). Food Packaging and Shelf Life 11: 58-66.

Griga, M., Novák, F.J. Pea (Pisum sativum L.)[M]. 1990. Springer Berlin Heidelberg: 65-99. ISBN: 978-3-642-74450-1.

Guindon, F., Cazzola, F., Palacios, T., Gatti, I., Cointry, E. 2021. Biofortification of pea (Pisum sativum L.): A Review~~[J]~~. Journal of the Science of Food and Agriculture, 101(9): 1-11.

Ishaq, S., Rathore, H.A., Masud, T., Ali, S. 2009 . Influence of Post Harvest Calcium Chloride Application, Ethylene Absorbent and Modified Atmosphere on Quality Characteristics and Shelf Life of Apricot (*Prunus armeniaca L.*) Fruit during Storage. Pakistan Journal of Nutrition 8: 861-865

Jin, Z.Q., Zhang, J.S., Lin, X.Y. (2007). Study on Water Loss Rate and Decay of Strawberry by NMR and MRI~~[J]~~. Food sicence, 28(8): 108-111.

Kader, A.A. 1992. Postharvest Biology and Technology: An Overview, and Modified Atmosphere During Transport and Storage. In: Kader, A.A., (Ed.), Postharvest Technology of horticultural crops, University of California Pub 3311: 85-92.

Kumar, N. 2016. Preservative potential of cumin essential oil for Pisum sativum L. during storage~~[J]~~. Journal of Plant Protection Research 56(2): 203-210.

Kano, Y., Fukuoka, N. 1994 . Varietal differences in the occurrence of hollowing and lignification of parenchymatous cells in the roots of Japanese radish~~[J]~~.Japan Soc Hort Sci 62(4) : 801-809.

Liu, Z.L., Li, L., Luo, Z.S., Zeng, F.F., Jiang, L., Tang, K.C. 2016. Effect of brassinolide on energy status and proline metabolism in postharvest bamboo shoot during chilling stress. Postharvest Biology and Technology 111: 240-246.

Melida, H., Encina, A., Largo, Gosens, A. 2015 . Ectopic lignification in primary cellulose□deficient cell walls of maize cell suspension cultures[J]. Journal of Integrative Plant Biology (4): 357-372.

Maekawa, E., Ichizawa, T., Koshijima, T. 1989 . An Evaluation of the Acid-Soluble Lignin Determination in Analyses of Lignin by the Sulfuric Acid Method[J]. Journal of Wood Chemistry & Technology 9(4): 549-567.

Nasef, I.N., El-Seifi, S.K., Hassan, M.A., El-Bassiouny, R.E.I., Elwan, M.W.M. 2018. Effect of Modified Atmosphere Packaging on Storability, Shelf Life and Quality of Snow Peas. Hortscience Journal of Suez Canal University 7(2): 81-90.

Pariasca, J.A.T., Miyazaki, T., Hisaka, H., Nakagawa, H. and Sato, T. 2001. Effect of modified atmosphere packaging (MAP) and controlled atmosphere (CA) storage on the quality of snow pea pods (*Pisum sativum L. var. saccharatum*). Postharvest Biology and Technology, 21(2): 213-223.

Peter, G., Neale, D. 2004. Molecular basis for the evolution of xylem lignification[J]. Current Opinion in Plant Biology 7(6):737-742.

Rissolo, A., Vanoli, M., Bianchi, G., Zanella, A., Grassi, M., Torricelli, A., Spinelli, L. 2014. Relationship between texture sensory profiles and optical properties measured by time -resolved reflectance spectroscopy during post-storage shelf life of ‘braeburn’apples[J]. Journal of Horticultural Research 22(1): 113-121.

Shi, M., Zhang, Z., Yu, S.,Wang, K., Gilbert, R.G., Gao, Q. 2014. Pea starch (*Pisum sativum L.*) with slow digestion property produced using β-amylase and transglucosidase[J]. Food Chemistry 164: 317-323.

Song, H., Yue, Na, W.U., Mei, L. 2010 . Quantitative determination of total free-Amino Acid in Nervilia fordii (Hance) Schltr. by Ninhydrin Colorimetric Method[J].

Chinese Journal of Information on Traditional Chinese Medicine 53(7): 1849-1859.

Terrett, O.M., Dupree, P. 2019. Covalent interactions between lignin and hemicelluloses in plant secondary cell walls - ScienceDirect[J]. Current Opinion in Biotechnology 56:97-104.

Zhu, N., Zhao, C., Wei, Y., Sun, C., Wu, D., Chen, K.S. 2021. Biosynthetic labeling with 3-O-propargylcaffeyl alcohol reveals in vivo cell-specific patterned lignification in loquat fruits during development and postharvest storage[J]. Horticulture Research 8: 61-78.

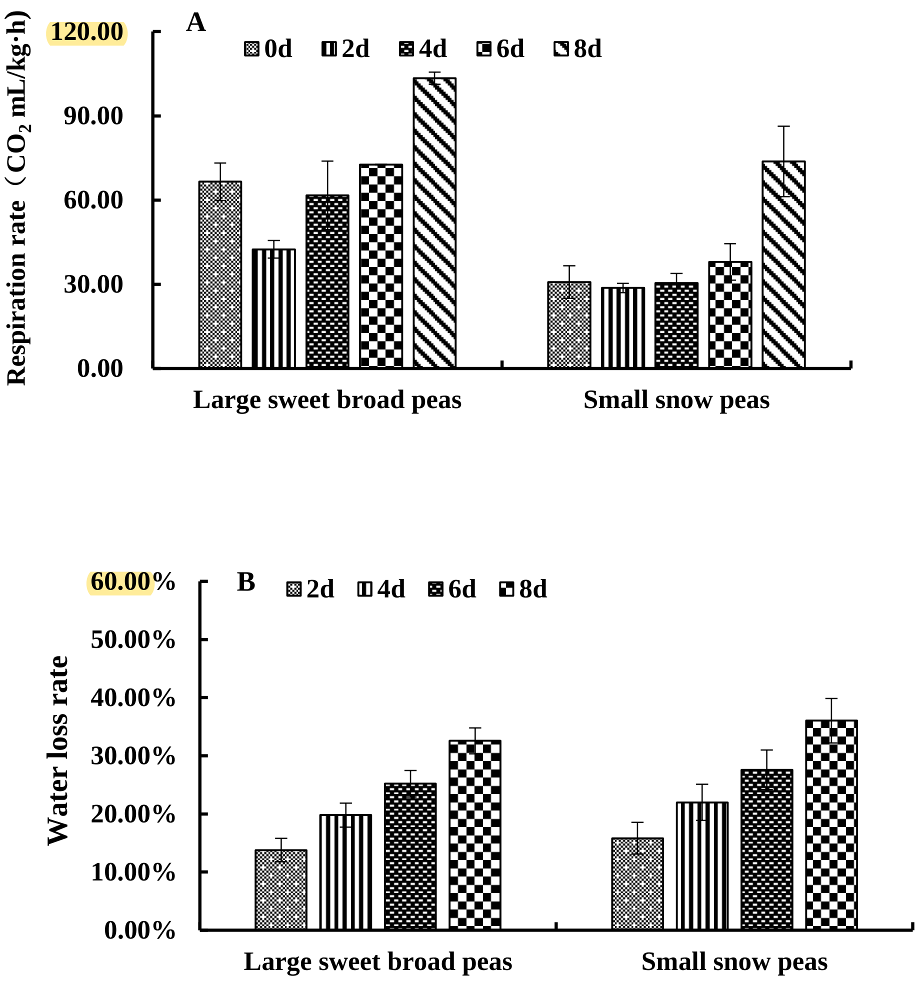

Fig 1. Changes of respiration rate (A) and water loss rate (B) of two varities of sugar snap pods

during storage

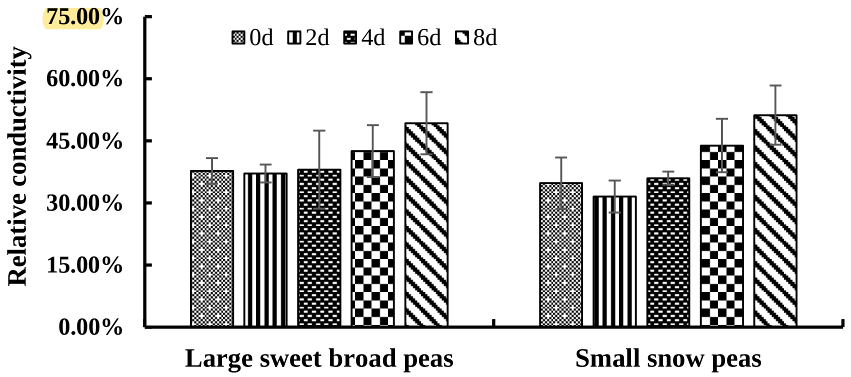

Fig 2. Changes in relative conductivity of two varieties of snow pea during storage

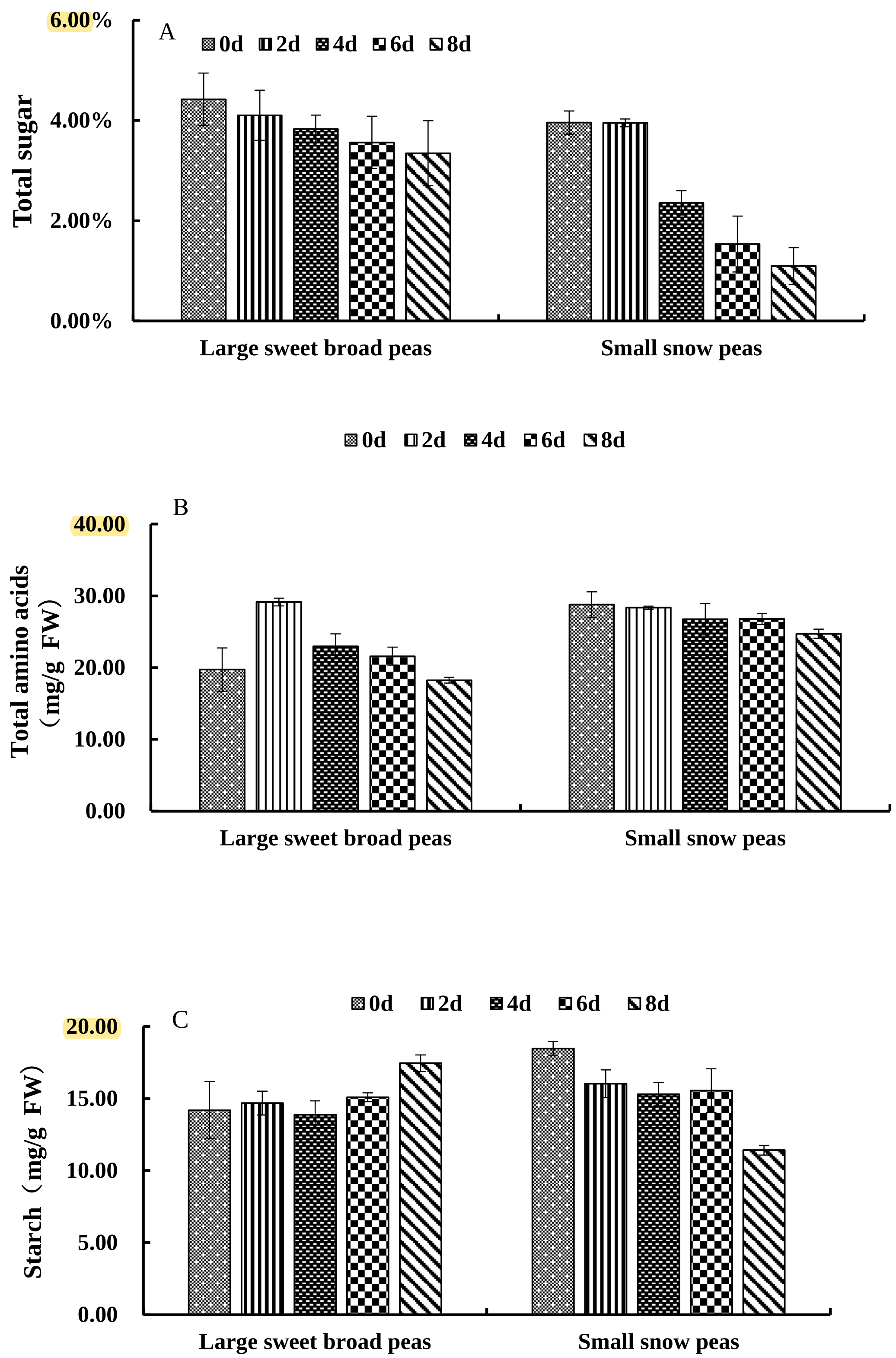

Fig 3. Changes in total sugar (A), total amino acid (B), and total starch (C) content

in two varities of snow pea during storage

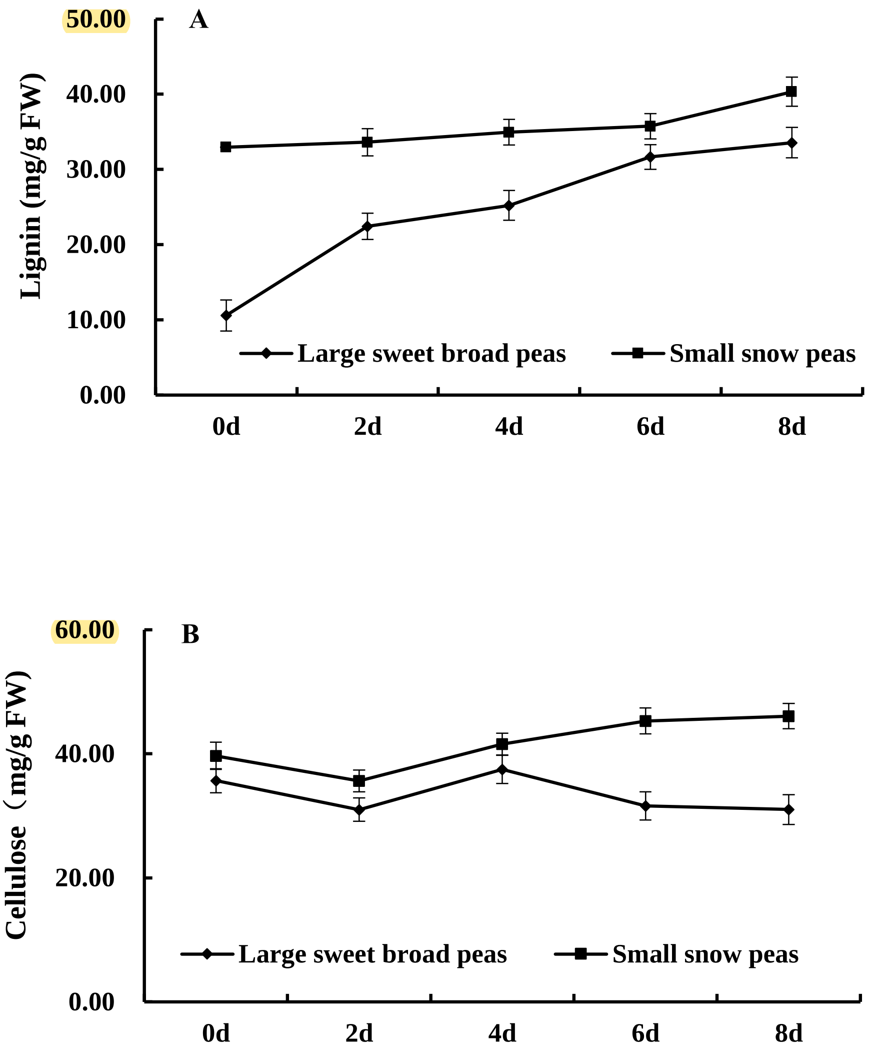

Fig 4. Changes of lignin,cellulose contents of two kinds of snow pea during storage

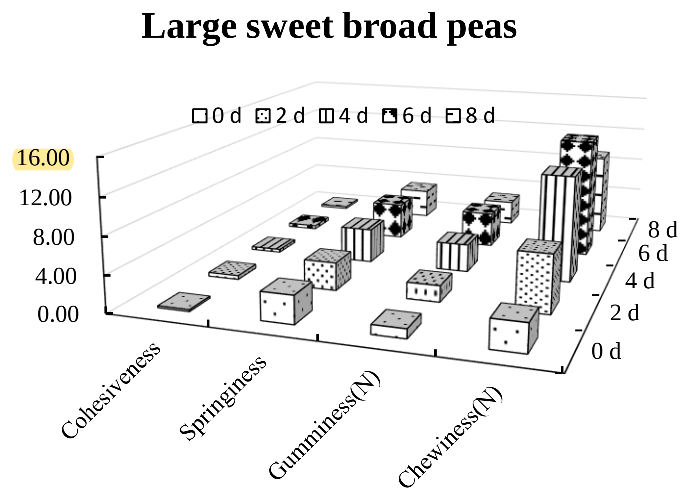

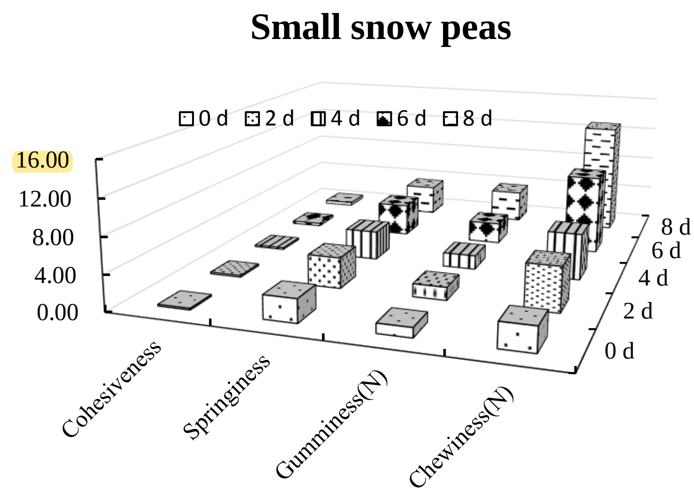

Fig 5. Changes of texture characteristics content of legumes vegetables during storage

Click here to access/download
Supporting Information
language polishing statement by Ping Liu.doc
